# Comparison of Biowaste Fillers Extracted from Fish Scales and Collagen on the Mechanical Properties of High-Density Polyurethane Foams

**DOI:** 10.3390/polym16192825

**Published:** 2024-10-06

**Authors:** Zodidi Obiechefu, Stanley Chibuzor Onwubu, Deneshree Naidoo, Thabang Hendrica Mokhothu, Phumlane Selby Mdluli

**Affiliations:** 1 Chemistry Department, Durban University of Technology (DUT), Durban 4001, South Africa; zodidi68@gmail.com (Z.O.); deneshree.naidoo3@gmail.com (D.N.); thabangm1@dut.ac.za (T.H.M.); 2 Health Platform, Advanced Materials Division, Mintek, Randburg 2194, South Africa; phumlanem@mintek.co.za; 3 Faculty of Applied Science, Durban University of Technology, Durban 4001, South Africa

**Keywords:** biowaste fillers, fish scales, collagen, polyurethane foams, mechanical properties, sustainability

## Abstract

The utilization of biowaste fillers in the development of high-density polyurethane (PU) foams has gained significant attention due to environmental and economic benefits. This study investigates the mechanical properties of PU foams reinforced with biowaste fillers extracted from fish scales (FS) and fish scale-derived collagen (FSC). The fish scales and collagen were characterized for their composition and integrated into PU foams at various loadings. Mechanical properties such as tensile strength, hardness, and density were evaluated. ANOVA was used to analyze the mean values. Bonferroni tests were used to identify differences between the filler materials (α = 0.05). The tensile strength increases with an increase in filler content for both FS (59.48 Kpa) and FSC (65.43 Kpa). No differences were observed between FS and FSC in tensile strength. Significant differences were observed between the FS and FSC in both hardness and density (*p* < 0.001). The results demonstrated that both fillers enhanced the mechanical properties of PU foams, with collagen-reinforced foams showing superior performance. This suggests that collagen and fish scales can be effective biowaste fillers for developing environmentally friendly PU foams with enhanced mechanical properties.

## 1. Introduction

In the realm of polymeric materials, polyurethane (PU) is considered very different and versatile when compared to its contemporary polymers [1]. PU polymer is formed by the reaction between diisocyanate and polyester diol [2,3], which is vital in the polyurethane industry. Das and Mahanwar [1] note that the simplicity of the production and the excellent properties that they provide have made PU one of the most sought-after polymers. For example, the thermal conductivity coefficient (λ) of PU foams measured in the ranges from 0.018 to 0.025 W·m^−1^·K^−1^ makes it highly sought after in diverse applications, including construction, industrial insulation, and household appliances [4]. Also, PU is widely utilized in various technical applications because of its high tensile strength, chemical resistance, ease of processing, and excellent mechanical properties [1]. However, traditional components in PU formation, such as polyols, are derived from petrochemical sources, raising concerns about their environmental impact and sustainability [5]. In response to these concerns, researchers are now using innovative green materials that rely on natural resources [6]. Among the renewal materials, vegetable oil, which is plentiful and widely used, is the primary renewable source for producing raw materials for PU products [4,7]. Hence, numerous researchers have created polyols from various vegetable oils, including soybean oil [8], castor oil [9], and rapeseed oil [10], among others, to develop a new type of bio-based polyurethane foam [11,12]. However, these natural biobased PU foams reportedly have a relatively low mechanical strength [13]. To address these drawbacks, there is a growing interest in developing bio-based PU foams that incorporate natural fillers derived from renewable sources. It has been suggested in the literature that the incorporation of different kinds of organic and inorganic fillers may enhance the mechanical properties of PU composite foams [4]. For instance, inorganic fillers, such as nanoclay [14], expandable graphite [15], silica [16], talc [17], or polyhedral oligosilsesquioxanes (POSS) [16,18], have been widely reported in enhancing the mechanical properties of PU foams.

Nevertheless, the need for a biodegradable filler material that aligns with the concept of circular and green chemistry has led to research in the use of natural fillers from agro-waste. Husainie et al. [19] report that enhancing the mechanical properties of PU foams with natural fillers can broaden their application range and improve their functionality. Moreover, incorporating biowaste fillers into PU foams could address both environmental and performance objectives [20]. This is supported by numerous studies, such as cellulose nanocrystals [21] and wheat straw lignin [22], that show the enhancement of mechanical properties of PU foams with natural fillers. Biowaste fillers, particularly extracted from fish scales, offer a promising solution and represent a step towards circular economy practices, where waste materials are repurposed to create value-added products [23,24]. Moreover, utilizing raw materials from biobased sources enables the introduction of biogenic carbon into the product life cycle. This could ultimately result in a carbon footprint reduction [25]. Fish scales are an abundant byproduct of the fishing industry, making them an ideal filler material for sustainable material development. Fish scales are composed of collagen fibers, which are known for their high strength, stiffness, and toughness [26]. Consequently, fish scales have been used in enhancing the properties of PU foams. Zieleniewska et al. [27], for example, synthesized PU composite foams enhanced with eggshells. The incorporation of eggshells into the PU matrix improved the mechanical properties, reduced water uptake, and increased dimensional stability in selected aqueous media. Similarly, Ref. [28] reported that the addition of fish scale powder (such as 0.5–1 wt%) enhances both the tensile and elongation properties of PU compared to the unreinforced PU foam. The authors attributed the improved strength to the collagen constituents of the fish scale powder. Additionally, collagen has been used in various biomedical and industrial applications due to its biocompatibility and mechanical properties [29].

Although studies have investigated the reinforcing properties of various bio-based fillers, such as fish scales and collagen, the direct comparison between fish scales and collagen as fillers in PU foams is lacking. While both materials have shown potential individually, as mentioned by [29,30], their relative effectiveness in enhancing PU foam properties has not been comprehensively studied. This research aims to provide a detailed comparison of PU foam reinforced with fish scale powder with that of fish scale-derived collagen at standard temperature. The novelty of this research lies in its dual approach, which not only assesses the performance of two bio-based fillers simultaneously but also investigates their optimal weight concentrations for enhanced mechanical properties. We envisaged that the direct comparison of the reinforcing properties could help provide a clear understanding of how these fillers influence the mechanical performance of PU foams, thereby guiding future material development efforts. Furthermore, the successful incorporation of these natural fillers could broaden the application range of PU foams, making them more suitable for diverse industrial and consumer products. The purpose of this study is to compare and analyze the mechanical and structural properties of fish scales and collagen as fillers in PU foams. Different weight concentrations of fish scale powder synthesized through the milling process (0.5 wt% and 1 wt%) and fish scale-derived collagen (5%, 10%, and 15%) were dropped into PU foams. The mechanical properties were studied using tensile strength, hardness, and density. This study hypothesizes that there will be differences in the enhancing properties of fish scales (FS) and fish scale-derived collagen (FSC).

## 2. Materials and Methods

### 2.1. Collection and Pretreatment of Fish Scales

Fish scales were sourced from local markets within the Durban and Chatsworth areas. After collection, the fish scales were subjected to pre-treatment. The treatment entailed washing and disinfecting the fish scales. The collected fish scales were soaked and disinfected thoroughly with distilled water and 5 mL bleach and stored at −20 °C until use. Fish scales were then defrosted to room temperature and followed by rinsing with deionized water. The extraction of collagen and composite preparation will be carried out following different steps.

### 2.2. Preparation of Powdered Fish Scales

The fish scales were soaked in 0.1 N NaOH for two days to eliminate non-collagenous proteins and pigments. After this treatment, they were rinsed with distilled water and then sun-dried for 2–3 weeks. Once dried, the fish scales were ground in a 250 mL bowl at 500 rpm for one hour using a planetary ball mill (Retsch^®^ PM 100) containing thirty stainless steel balls, each with a diameter of 10 mm.

### 2.3. Extraction of Collagen from Fish Scales (Optimization)

**Figure 1:**
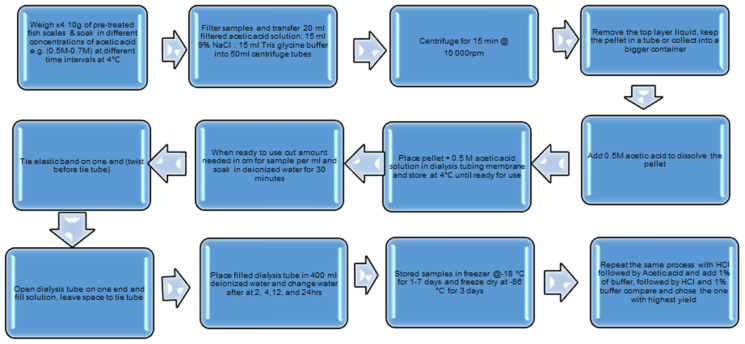
Extraction process of collagen from fish scales.

The collagen was prepared as illustrated in Figure 1.

### 2.4. Preparation and Testing of the Polyurethane Composites

Table 1 outlines the general formula used for preparing PUF-based composites. Flexible PUFs were created by mixing milled fish scale (FS) and collagen extracted from fish scale (FSC) separately with PUF using a mechanical stirrer. Various weight percentages of FS (from 0 to 1 wt%) and FSC (2.5 to 10 wt%) were added to the PU to produce composites with different reinforcement levels. The chemical components were measured to achieve a target density of 16–17 kg/m^3^. The experiment took place in a Foam Laboratory equipped with a manual mixing apparatus, including a stirrer with two speeds (600 and 1300 rpm) powered by a 7.5 HP motor, ensuring efficient mixing. The chemical ingredients were manually measured and added to a large 3 L container. The materials followed a standard PU recipe. The preparation of PUF-based composites involved two main steps. First, polyol and other ingredients such as methyl chloride, catalysts, additives, and water were weighed and placed in the 3 L container. The FS was then added as a filler and stirred for 20–30 s. In the second step, specific amounts of toluene di-isocyanate (TDI) were weighed separately in a 500 mL cup and added to the stirred polyol mixture, followed by another 10–15 s of stirring before the mixture began to cream. This mixture was immediately transferred into a 305 × 235 × 305 mm mold, where cream and rise times were recorded. After the polyurethane foam fully rose, it was transferred to a 70 °C oven to cure for 10 min. This process was repeated five times with different amounts of filler added to the standard formulation. The cured foam was then cut using a bandsaw.

### 2.5. Characterization of Physical Properties of Pu-Based Composites

The prepared PU foams were subjected to mechanical testing, including density and hardness tests, to evaluate the impact of the fillers on the foam properties. A 2 kN Instron was utilized for the tensile test, which was conducted in accordance with SABS 640–1976 Section 6.8 regulations. The SANS 883:2009 was followed for conducting the elongation at break test. The item was shaped like a dog bone and was 150 × 27 × 17 mm. The gauge length was set at 45 mm, and the machine speed was set to 500 mm/min. Tensile strength findings were recorded in kPa, and the test was maintained until the sample burst. To obtain an average value for statistical analysis, five samples underwent tensile and elongation testing.

For density measurements, samples sized 100 × 100 × 50 mm were used. A steel ruler with millimeter markings and an accuracy of 1 mm was employed to measure the object’s length, width, and height. The sample’s mass was determined using a scale with a sensitivity of 0.1 g. The results were analyzed to compare the performance of fish scales and collagen-reinforced PU foams. Density was standardized as all samples were cut from the bottom of the rise profile. The mass per unit volume of a sample was measured and calculated using the formula: D = M/V × 1000

D is density (kg/m^3^), M is mass (kg), and V is volume (m^3^).

Hardness was conducted using the hardness Vickers type at room temperature. The scale ranges from 0 to 100, where 100 represents overall hardness and 0 represents overall penetration. Ten measurements were taken for each sample. The final compliance value was determined according to the ASTM D2240 standard [31].

### 2.6. Characterization of FS and FSC Reinforced PU Foams

The Perkin Elmer Universal ATR (Connecticut, USA) was employed to identify the functional groups in the prepared MFS and FSC samples. A background check was performed before scanning. Small amounts of the prepared sample powders were then placed in a sample holder and scanned within the 400 to 4500 cm^−1^ range at a resolution of 4 cm^−1^.

### 2.7. Data Analysis

Statistical analyses were conducted to determine the significance of the observed differences. Using the Statistical Package for the Social Sciences (SPSS, IBM, Chicago, IL, USA, version 29), a one-way analysis of variance (ANOVA) was performed to examine the mean differences in tensile, hardness, and density at a significance level of α = 0.05.

## 3. Results and Discussion

### 3.1. Tensile Strength

#### 3.1.1. Tensile Strength of Reinforced PU Foam with FSC and FS at Different Concentrations

**Figure 2:**
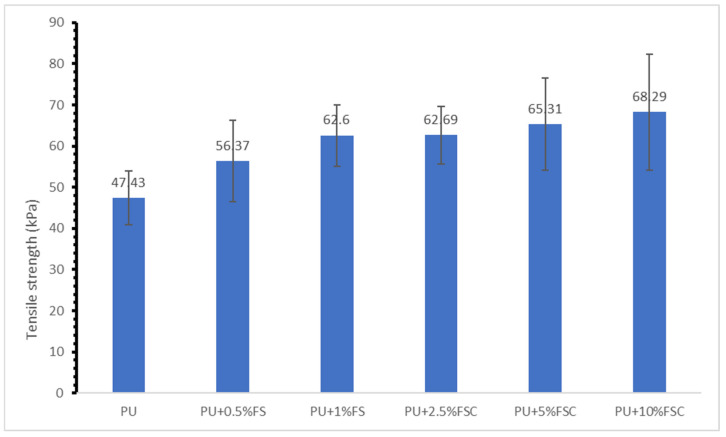
Tensile strength of reinforced PU foams with different concentrations of FSC and FSC.

The tensile strength of polyurethane (PU) foams reinforced with different concentrations of fish scales (FS) and fish scale collagen (FSC) is shown in Figure 2. The results indicate that the incorporation of both FS and FSC enhances the tensile strength of the PU foam compared to the neat PU foam. The neat PU foam exhibited a tensile strength of approximately 47.43 kPa. It was observed that the addition of 0.5 g and 1 g of reinforcement fish scale powder improved the tensile strength of the polyurethane foam composites and resulted in improved physical properties. The addition of 0.5% FS increased the tensile strength to 56.37 kPa. The further increase in FS to 1% resulted in a tensile strength of 62.6 kPa. Likewise, the addition of 2.5%, 5%, and 10% of reinforcement fish scale powder also improved the tensile strength of the polyurethane foam composites and resulted in improved physical properties. The addition of 2.5% FSC resulted in a tensile strength of 62.69 kPa, slightly higher than that of 1% FS. Incorporating 5% FSC increased the tensile strength to 65.31 Kpa. The highest tensile strength observed was 68.29 kPa with the addition of 10% FSC.

The results demonstrate that both fish scales and fish scale collagen significantly enhance the tensile strength of PU foams. This improvement can be attributed to the reinforcing effect of the fillers, which contribute to the overall mechanical integrity of the composite material. Fish scales contain collagen fibers known for their high strength and stiffness. The observed increase in tensile strength with FS addition is consistent with previous studies that highlighted the reinforcing potential of biowaste fillers in polymer matrices [28]. Moreover, as FS content increases from 0.5% to 1%, the tensile strength shows a noticeable enhancement, suggesting that FS effectively reinforces the PU matrix. The results suggest a positive correlation between filler content and tensile strength for both FS and FSC, implying that as filler content increases, the tensile strength increases. Collagen, being a major component of fish scales, offers superior mechanical properties, including high tensile strength and biocompatibility. The increasing trend in tensile strength with higher FSC content aligns with findings from other studies that utilized collagen as a reinforcement in composite materials [29,32]. The highest tensile strength observed at 10% FSC (68.29 kPa) indicates that collagen significantly contributes to the mechanical robustness of the PU foam, likely due to its inherent structural properties that enhance stress distribution and load-bearing capacity [33].

#### 3.1.2. Comparison of the Tensile Strength between FSC and FS

The one-way ANOVA, mean ±SD, standard error (SE), and post hoc results are illustrated in Table 2 Significant differences were found between tensile strength values for the PU foams reinforced with FS (fish scale) and FSC (fish scale-derived collagen). The unreinforced (neat) had the lowest tensile strength (47.43 ± 6.53 KPa), whereas FSC-reinforced PU foams had the highest tensile strength (65.43 ± 9.95 KPa). The post hoc test shows that FSC was significantly higher than those of the neat (*p* = 0.029). No significant differences were found between the tensile strength values of FS and FSC (*p* > 0.05). Likewise, there were no significant differences between the FS and neat (*p* < 0.05). The significant increase in tensile strength when FS and FSC are incorporated demonstrates the reinforcing potential of these natural fillers. Neat PU tends to have lower mechanical strength in comparison, as polymers without reinforcement generally exhibit less resistance to tensile stress. Incorporation of FS as a filler in polymer matrices often leads to improvements in mechanical properties such as tensile strength. This is supported by several studies that have highlighted the reinforcing effect of natural fillers in composite materials. For instance, Kuciel et al. reported that natural fillers, including FS, enhance the tensile strength of polymeric materials due to their ability to distribute stress more effectively throughout the polymer matrix [34].

FSC provides superior tensile strength compared to FS, which can be explained by the structural integrity and strong bonding properties of collagen. Collagen has been widely recognized for its high mechanical strength, making it an ideal candidate for reinforcement in polymer matrices. Rezvani Ghomi et al. [29] found that collagen-based fillers in composite materials significantly improve tensile strength due to strong hydrogen bonding and the inherent mechanical properties of collagen. Also, the superior tensile strength of FSC may be attributable to the higher purity and better dispersion of collagen compared to the whole fish scales. Previous studies have reported similar trends with other natural fillers. For instance, the addition of cellulose nanocrystals and lignin also resulted in enhanced mechanical properties of PU foams [21,22].

**Figure 3:**
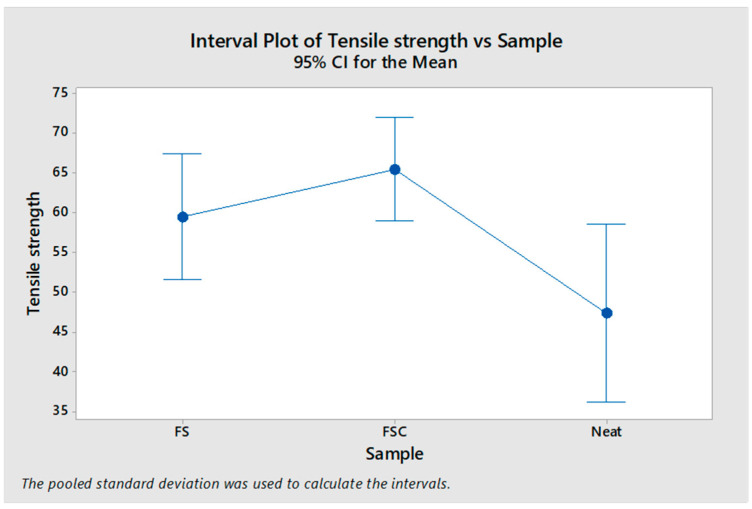
Differences in mean tensile strength (Kpa) of PU foams reinforced with FS and FSC.

Figure 3 further illustrates the differences in the mean value of the tensile strength of the PU foam. The interval plot indicates that both FS and FSC significantly improve the tensile strength of polyurethane compared to neat PU. FSC, in particular, shows the highest tensile strength (65.43 ± 9.95 KPa), which is consistent with the literature indicating that collagen-based fillers enhance the mechanical properties of polymers. The confidence intervals further suggest that while there is some variability in the data, the trend of improved tensile strength with natural fillers is clear.

### 3.2. Hardness Assessment

#### 3.2.1. Hardness of Reinforced PU Foam with FSC and FS at Different Concentrations

**Figure 4:**
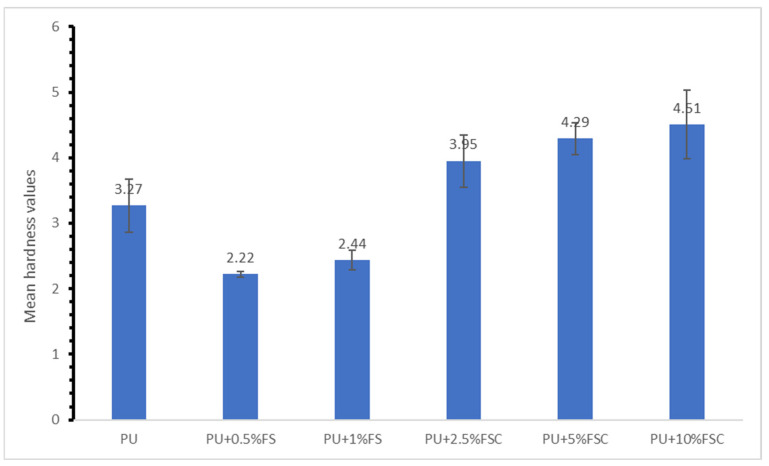
Hardness of reinforced PU foams with different concentrations of FSC and FSC.

Figure 4 illustrates the mean hardness values of polyurethane (PU) foams reinforced with different concentrations of fish scales (FS) and fish scale collagen (FSC). The results indicate that the incorporation of FSC significantly enhances the hardness of PU foams, whereas the addition of FS results in a decrease in hardness compared to neat PU foam. The results suggest different impacts on the hardness of PU foams depending on the type and concentration of the filler used. The addition of fish scales (0.5% and 1%) leads to a decrease in hardness compared to the neat PU foam. This reduction might be attributed to the poor dispersion of fish scales within the PU matrix or the inherent properties of the scales themselves. The literature supports that the mechanical properties, including hardness, can be adversely affected if the filler-matrix compatibility is not optimal [28]. The lower hardness values with FS might indicate less effective reinforcement compared to FSC. The inclusion of FSC at varying concentrations (2.5%, 5%, and 10%) shows a significant increase in hardness, with the highest value observed at 10% FSC (4.51). Collagen is known for its high mechanical strength and excellent compatibility with various polymer matrices, which can explain the improved hardness values [29]. The increased hardness suggests better stress transfer and reinforcement within the PU matrix. Similar trends have been observed in other studies where collagen or other biopolymers were used as fillers in composites, leading to enhanced mechanical properties, including hardness [30].

The contrasting effects of FS and FSC on hardness underscore the importance of filler type and its interaction with the polymer matrix. While FS may contain beneficial collagen fibers, the overall structure and composition might not be as conducive to enhancing hardness as pure collagen. Previous research on bio-based fillers such as cellulose nanocrystals and lignin also indicates that the effectiveness of reinforcement strongly depends on the filler characteristics and their dispersion within the polymer [21,22]. The mean hardness values show a clear distinction between the effects of FS and FSC on the PU foams. The standard deviations, while relatively small, highlight the consistency of the measurements. The significant increase in hardness with FSC addition demonstrates its potential as an effective reinforcement material for enhancing the mechanical properties of PU foams. In conclusion, while the incorporation of fish scales leads to a reduction in hardness, fish scale collagen significantly enhances the hardness of PU foams. This highlights the superior reinforcing effect of collagen, likely due to its excellent mechanical properties and compatibility with the polymer matrix. Future research should focus on optimizing the dispersion and concentration of such bio-based fillers to maximize their reinforcing potential in sustainable composite materials.

#### 3.2.2. Comparison of the Hardness between FSC and FS

The one-way ANOVA, mean ±SD, standard error (SE), and post hoc results are illustrated in Table 3. Significant differences were found in the hardness value of PU foams reinforced with FS (fish scale) and FSC (fish scale-derived collagen). FS had the lowest hardness value (2.33 ± 0.16), whereas FSC-reinforced PU foams had the highest hardness value (4.25 ± 0.38). The post hoc test shows that FSC was significantly higher than those of the neat (*p* = 0.001) and FS (*p* < 0.001). The hardness value measured for the neat was significantly higher than FS (*p* = 0.003).

The data clearly show that the addition of fish scales (FS) leads to a decrease in hardness, whereas adding both fish scales and collagen (FSC) results in a substantial increase in hardness compared to the control. The decrease in hardness upon adding fish scales can be attributed to the brittle nature of fish scales, which may not effectively bond with the polymer matrix. Studies have shown that natural fillers, such as fish scales, can sometimes reduce mechanical properties due to poor compatibility between the filler and the polymer matrix. For instance, poor dispersion of the scales can lead to weak points within the composite, lowering overall hardness [35]. Collagen, being a natural protein, can provide better adhesion within the polymer matrix. The literature indicates that collagen fibers can form strong interfacial bonding with polymers, improving both mechanical strength and hardness [33]. Additionally, collagen’s fibrous structure can distribute stress more evenly, resulting in a harder, more durable composite [36].

### 3.3. Density

#### 3.3.1. Density of Reinforced PU Foam with FSC and FS at Different Concentrations

**Figure 5:**
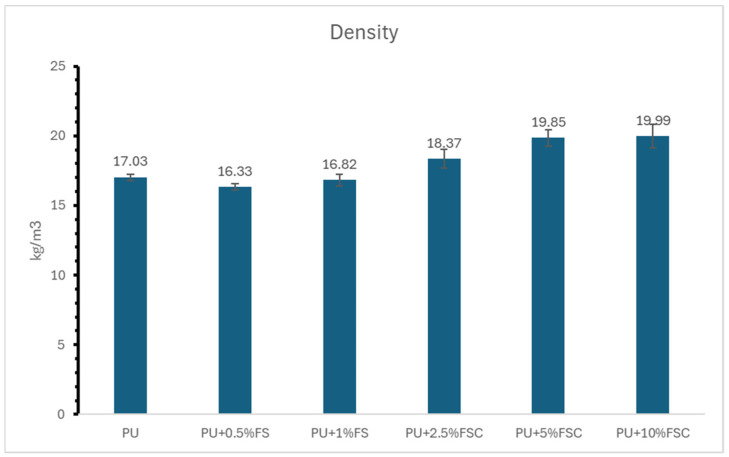
Density of reinforced PU foams with different concentrations of FSC and FSC.

Figure 5 illustrates the density values of polyurethane (PU) foams reinforced with different concentrations of fish scales (FS) and fish scale collagen (FSC). The results indicate a trend where the incorporation of FSC increases the density of the PU foams, whereas the addition of FS results in a slight decrease or marginal increase in density compared to the neat PU foam. The results suggest different impacts on the density of PU foams depending on the type and concentration of the filler used. The addition of fish scales (0.5% and 1%), for example, results in a slight decrease or marginal increase in density compared to the neat PU foam, respectively. This indicates that the fish scales do not significantly alter the density of the PU foam at these concentrations. The decrease in density could be due to the partial replacement of the denser PU matrix with the lighter fish scales. This observation aligns with other studies that report minimal changes in density when low concentrations of natural fillers are used [28]. The inclusion of FSC at varying concentrations (2.5%, 5%, and 10%) shows a significant increase in density, with the highest value observed at 10% FSC (19.99 kg/m^3^). The increase in density with FSC addition is likely due to the higher density of collagen compared to the PU matrix. Collagen’s dense molecular structure contributes to the overall increase in the composite density [29]. Similar trends have been observed in other studies where the addition of dense fillers like cellulose nanocrystals resulted in increased density of the composite materials [21].

#### 3.3.2. Comparison of the Density between FSC and FS

The one-way ANOVA, mean ±SD, standard error (SE), and post hoc results are illustrated in Table 4. Significant differences were found in the hardness value of PU foams reinforced with FS (fish scale) and FSC (fish scale-derived collagen). FS had the lowest hardness value (2.33 ± 0.16), whereas FSC-reinforced PU foams had the highest hardness value (4.25 ± 0.38). The post hoc test shows that FSC was significantly higher than those of the neat (*p* = 0.029). No significant differences were found between the tensile strength values of FS and FSC (*p* > 0.05). Likewise, there were no significant differences between the FS and neat (*p* < 0.05). The significant increase in tensile strength when FS and FSC are incorporated demonstrates the reinforcing potential of these natural fillers.

The contrasting effects of FS and FSC on density highlight the different physical properties and dispersion behaviors of these fillers. While FS may provide some reinforcement, its impact on density is less pronounced compared to FSC. Previous research on bio-based fillers such as lignin and nanoclay also indicates that the effectiveness of reinforcement and its impact on density strongly depends on the filler characteristics and their interaction with the polymer matrix [22]. The mean density values show a clear distinction between the effects of FS and FSC on the PU foams. The standard deviations, while small, indicate consistent and reliable measurements. The significant increase in density with FSC addition demonstrates its potential as a high-density reinforcement material for enhancing the mechanical properties of PU foams. In conclusion, the incorporation of fish scales results in a slight decrease or marginal increase in the density of PU foams, whereas fish scale collagen significantly increases the density. This suggests that FSC, due to its higher density and structural properties, provides a more substantial reinforcement effect, making it a promising candidate for developing high-performance PU composites. Future research should explore the optimization of filler content and processing techniques to maximize the benefits of these bio-based fillers in sustainable composite materials.

### 3.4. FTIR Spectrum of Reinforced PU Foam

**Figure 6:**
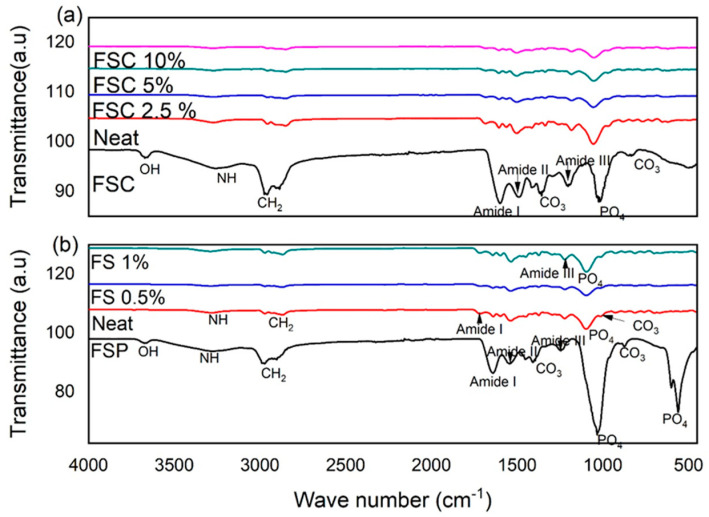
FTIR spectra of (**a**) fish scale-derived collagen and (**b**) fish scale powder.

Figure 6 shows the FTIR (Fourier Transform Infrared) spectra of different PU composites with varying concentrations of fish scales (FS) and fish scale collagen (FSC). FTIR spectroscopy is used to identify the functional groups present in the samples and to understand the chemical interactions within the composites. The spectrum for neat PU shows characteristic peaks at around the region of 3325 cm^−1^ associated with the N-H stretching vibrations, indicating urethane linkage. The stretching vibrations in the range of 2940 cm^−1^ and 2860 cm^−1^ due to C-H stretching vibrations were observed. In the region of 1730 cm^−1^ C=O stretching vibrations were observed due to the urethane carbonyl group. In the region of 1220 cm^−1^ C-O-C stretching vibrations were observed. The spectra for PU + 0.5% FS and PU + 1% FS show similar peaks to neat PU, with slight shifts and changes in intensity. A slight decrease in the intensity of the N-H stretching and C=O stretching peaks is observed, suggesting some interaction between the PU matrix and FS. The spectra for PU + FSC composites show more pronounced changes; a slight shift in the N-H stretching peak to lower wavenumbers indicates hydrogen bonding between the PU matrix and FSC. The C=O stretching peak becomes broader and shifts slightly, suggesting interactions between the carbonyl groups of PU and the collagen structure. Additional peaks around 1650 cm^−1^ (amide I band) and 1550 cm^−1^ (amide II band) become more prominent with increasing FSC content, confirming the presence of collagen.

Interaction between PU and FS, the FTIR spectra indicate that the FS particles do not significantly alter the chemical structure of the PU matrix at low concentrations (0.5% and 1%). Similar observations were reported by Naidoo et al. [28], where the incorporation of low concentrations of natural fillers resulted in minimal changes to the polymer matrix. Regarding the interaction between PU and FSC, the shifts in the N-H and C=O stretching peaks suggest strong hydrogen bonding between the PU matrix and FSC. The broadening and shifting of peaks confirm the successful incorporation of FSC into the PU matrix, enhancing its structural integrity and mechanical properties. This interaction can enhance the mechanical properties of the composites by improving the compatibility between the matrix and the filler. A previous study by Rezvani Ghomi et al. [29] reported similar findings where collagen-reinforced polymers showed improved mechanical properties due to strong interfacial bonding, supported by the study result in Figure 2.

## 4. Discussion

This study investigated the effects of incorporating fish scales (FS) and fish scale-derived collagen (FSC) into polyurethane (PU) foams, focusing on tensile strength, hardness, density, and chemical interactions as characterized by FTIR spectroscopy. The results revealed that FSC significantly enhances the tensile strength and hardness of PU foams compared to FS and neat PU. Specifically, PU foams reinforced with FSC exhibited the highest tensile strength (65.43 ± 9.95 KPa) and hardness (4.25 ± 0.38), whereas FS did not show significant improvements over neat PU. The superior performance of FSC can be attributed to collagen’s inherent mechanical strength and its ability to form strong hydrogen bonds with the PU matrix, improving the overall mechanical properties [29]. This finding aligns with the literature indicating that collagen-based fillers can substantially enhance the mechanical properties of polymer composites due to their high strength and compatibility with polymer matrices [30,36].

In contrast, FS showed a decrease in hardness and only a slight increase in density, indicating that fish scales may not effectively reinforce PU foams as much as collagen. The reduction in hardness with FS could be due to poor dispersion and bonding within the PU matrix, a factor that has been noted in other studies on natural fillers [28,36].

FTIR analysis revealed significant chemical interactions between PU and FSC, as evidenced by shifts in the N-H and C=O stretching peaks and the appearance of amide bands. These interactions suggest that collagen forms strong hydrogen bonds and enhances compatibility with the PU matrix, thereby improving the mechanical properties of the composites [29]. The minimal changes observed with FS indicate that its effect on the chemical structure of PU is limited, consistent with findings that low concentrations of natural fillers have negligible effects on polymer matrices [28].

The findings of this study have several implications for the development of high-performance PU composites. First, the incorporation of FSC significantly improves the mechanical properties of PU foams, making them suitable for applications requiring high strength and durability. This enhancement could be particularly beneficial in industries such as automotive, aerospace, and construction, where material performance is critical. Utilizing FSC, a byproduct of the fish industry, represents a sustainable approach to composite material development. This aligns with the growing emphasis on bio-based and eco-friendly materials in material science and engineering [21]. Furthermore, the study highlights the importance of choosing the appropriate type and concentration of filler. While FS offers some benefits, FSC provides superior reinforcement, demonstrating the need for careful selection of fillers based on their properties and interactions with the polymer matrix [22]. Future research should investigate a broader range of FSC concentrations and combinations with other bio-based fillers. This is essential in order to reveal optimal conditions for maximizing mechanical properties. Furthermore, studies on the dispersion techniques and compatibility of various fillers with PU matrices are needed to address challenges related to poor dispersion and bonding, as observed with FS [28]. Additionally, assessing the long-term durability and performance of PU composites reinforced with FSC under various environmental conditions (e.g., moisture, temperature) would provide insights into their practical applications [36]. Finally, comparing the performance of FSC with other natural and synthetic fillers in different polymer matrices could help in identifying the most effective reinforcement materials for specific applications [21,30].

## 5. Conclusions

Biowaste materials from fish scales offer environmentally sustainable filler materials for various applications. The study comparative analyses the mechanical and structural properties of fish scales (FS) and collagen (FSC) as fillers in PU foams. Based on the findings, both FS and FSC effectively enhance the mechanical properties of PU foams, particularly the tensile strength. Comparatively, FSC offers superior enhancement than FS in the tensile, hardness, and density properties of PU foams. The improvement in mechanical properties was supported by the FTIR results showing strong hydrogen bonding between the PU matrix and FSC. This study conclusively suggests that the use of both FS and FSC in PU foams aligns with environmentally sustainable practices.

## Figures and Tables

**Table 1:** The general formula for preparing PUF-based composites.

Materials	Sample Groups by Weight %					
	Std	Std + 0.5 g Fish Scale Powder	Std + 1.0 g Fish Scale Powder	Std +2.5 Collagen Powder	Std + 5.0 g Collagen Powder	Std + 10 g Collagen Powder
POLYOL-1906	170.01	170.01	170.01	170.01	170.01	170.01
TDI-T80	123.79	123.79	123.79	123.79	123.79	123.79
WATER	9.81	9.81	9.81	9.81	9.81	9.81
CATALYSTMIX-TIN/MESAMOL	2.75	2.75	2.75	2.75	2.75	2.75
AMINE MIX-1906/B18	0.69	0.69	0.69	0.69	0.69	0.69
SILICONE-L620	3.11	3.11	3.11	3.11	3.11	3.11
BLOWING AGENT-METHYL CHLORIDE	29.01	29.01	29.01	29.01	29.01	29.01
COLOR STABILIZER-CS-15	3.01	3.01	3.01	3.01	3.01	3.01
FILLER-KULU POWDER	50.01	50.01	50.01	50.01	50.01	50.01
FISH SCALE POWDER	0	0.5	1			
COLLAGEN POWDER	0			2.5	5	10

**Table 2:** Tensile strength comparison between FS and FSC.

Tensile Strength	N	Mean	Std. Deviation	Std. Error	95% Confidence Interval for Mean		*p*-Value	Bonferroni Test
					Lower Bound	Upper Bound		
Neat	3	47.4333	6.53957	3.77563	31.1881	63.6785	0.030	0.242 ^a,b^
FS	6	59.4833	8.53109	3.48280	50.5305	68.4362		0.702 ^b,c^
FSC	9	65.4289	9.95256	3.31752	57.7787	73.0791		0.029 ^a,c^

FS = Fish scales; FSC = Fish scales derived collagen. a = Neat, b = FSC, c = FSC.

**Table 3:** Hardness value comparison between FS and FSC.

Hardness	N	Mean	Std. Deviation	Std. Error	95% Confidence Interval for Mean		*p*-Value	Bonferroni Test
					Lower Bound	Upper Bound		
Neat	3	3.2700	0.40596	0.23438	2.2616	4.2784	<0.001	0.003 ^a,b^
FS	6	2.3300	0.15505	0.06330	2.1673	2.4927		<0.001 ^b,c^
FSC	9	4.2511	0.38166	0.12722	3.9577	4.5445		0.001 ^a,c^

FS = Fish scales; FSC = Fish scales derived collagen. a = Neat, b = FSC, c = FSC.

**Table 4:** Density value comparison between FS and FSC.

Density	N	Mean	Std. Deviation	Std. Error	95% Confidence Interval for Mean		*p*-Value	Post Hoc
					Lower Bound	Upper Bound		
Neat	3	17.0333	0.22502	0.12991	16.4744	17.5923	<0.001	1.000
FS	6	16.5767	0.41089	0.16774	16.1455	17.0079		<0.001
FSC	12	19.4017	0.99394	0.28693	18.7701	20.0332		<0.001

FS = Fish scales; FSC = Fish scales derived collagen.

## Data Availability

The raw data supporting the conclusions of this article will be made available by the authors on request.
